# Surface Etching of 3D Printed Poly(lactic acid) with NaOH: A Systematic Approach

**DOI:** 10.3390/polym12081711

**Published:** 2020-07-30

**Authors:** Matthias Schneider, Nora Fritzsche, Agnieszka Puciul-Malinowska, Andrzej Baliś, Amr Mostafa, Ilko Bald, Szczepan Zapotoczny, Andreas Taubert

**Affiliations:** 1 Institute of Chemistry, University of Potsdam, D-14476 Potsdam, Germany; mattschn@uni-potsdam.de (M.S.); norafritzsche@gmx.de (N.F.); amr.shaffei@uni-potsdam.de (A.M.); ilko.bald@uni-potsdam.de (I.B.); 2 Faculty of Chemistry, Jagiellonian University, PL-30-387 Kraków, Poland; puciula@poczta.onet.pl (A.P.-M.); andrzejbalis@gmail.com (A.B.); zapotocz@chemia.uj.edu.pl (S.Z.)

**Keywords:** surface modification, sodium hydroxide etching, poly(lactic acid), 3D printing, roughness, wettability, erosion

## Abstract

The article describes a systematic investigation of the effects of an aqueous NaOH treatment of 3D printed poly(lactic acid) (PLA) scaffolds for surface activation. The PLA surface undergoes several morphology changes and after an initial surface roughening, the surface becomes smoother again before the material dissolves. Erosion rates and surface morphologies can be controlled by the treatment. At the same time, the bulk mechanical properties of the treated materials remain unaltered. This indicates that NaOH treatment of 3D printed PLA scaffolds is a simple, yet viable strategy for surface activation without compromising the mechanical stability of PLA scaffolds.

## 1. Introduction

Poly(lactic acid) (PLA) is an interesting and highly popular bio- and biodegradable material [1,2]. PLA is easily accessible on a large scale and its properties can be adjusted by proper choice of the (co)monomers and polymerization conditions yielding polymers with different mechanical properties and degradation profiles [3,4,5].

The advent of 3D printing has brought further interest to PLA because PLA can easily be printed using simple and low cost approaches such as fused deposition modelling (FDM) [6,7]. 3D printing is interesting for the general area of biomaterials development because, in principle, it may be possible to provide patient-specific implants on-site and on-demand [2]. One major restriction to directly using 3D printing of PLA implants in a clinical setting (apart from issues like safety at work) [8] is the fact that biological tissue does not respond well to plain, untreated PLA [8,9]. There is thus a need to modify and adjust the surface chemistry and morphology to render the implant (candidate) more biocompatible [10]. Clearly, these processes need to be simple, safe, and highly reproducible [11].

PLA offers a broad spectrum of properties (molecular weight and weight distribution, stereochemistry, comonomers) that can be adjusted rather easily during polymerization or after synthesis via blending, physical, or chemical modification. However, this must be done before filament production and requires additional equipment and laboratory steps [1,2,3,4,12,13].

Instead of altering the entire polymer prior to printing, surface modification after the printing process can be used as well. Surface treatment of a printed object enables the use of commercially available PLA filaments from the printer manufacturer. This filament is optimized in shape (precisely manufactured round filament) and matches the technical requirements of the printer. A good match of filament and printer is crucial and we have indeed observed (unpublished data) that PLA filaments from other suppliers behave differently while printing.

Several studies on the surface treatment and modification of PLA surfaces exist. However, according to Mohd Sabee et al. [11] many existing methods of PLA surface modification like coating, entrapment, chemical conjugation, radiation treatment, and photo-grafting are often expensive, time consuming, and sometimes difficult to perform.

Argentati et al. [14] used solvent casting to obtain poly(L-lactic acid) (PLLA) films with a thickness of 0.25 mm. These films were treated with an oxygen plasma to modify the surface properties without altering the bulk material. The plasma treatment had no effect on Young’s modulus and elongation at break. Differential scanning calorimetry (DSC) showed no changes in melting temperature and crystallinity. Scanning electron microscopy (SEM) showed the formation of a nanostructured surface and the static contact angle (CA) decreased from 90° to below 10° after plasma treatment. IR spectra showed no changes in the carbonyl band at 1756 cm^−1^ and in the C-O stretching band at 1080 cm^−1^. Thus, the authors concluded that O_2_ plasma treatment does not lead to chemical changes on the surface. It is however likely that polymer chain scission and chemical modification may have taken place but cannot easily be detected.

Inagaki et al. [15] used an Ar plasma for surface modification of PLA films. The Ar plasma treatments led to no changes in CA and chemical composition. Based on their data, the authors concluded that the PLA was degraded by the Ar plasma treatment and small molecules like CO_2_, CH_2_=CH_2_, CHO-CH_3_, and CO were eliminated. The authors also suggested that the recombination of radicals created by the plasma led to the same PLA molecular structure as was present in the original polymer. As a result, the polymer was claimed to degrade without a change in chemical composition and the treatment was thus deemed unsuitable for PLA surface modification to obtain –COOH and –OH groups and to increase wettability.

In contrast to plasma treatments, sodium hydroxide is cheap, readily available, and the hydrolysis reaction of PLA with NaOH is easy to perform. Consequently, several groups have studied the effects of NaOH treatment on PLA surfaces. Mohd Sabee et al. [11] used NaOH solution treatments for surface modification of PLA microspheres and found a change in the surface morphology of the particles upon treatment.

NaOH treatment was also investigated on poly(lactic-co-glycolic acid) by Croll et al. [16]. They found changes in the surface properties (especially decreased contact angles) even after short treatment time with low concentrations of NaOH. Yang et al. [17] reported that the treatment of PLA with NaOH improved the biocompatibility due to the resulting higher hydrophilicity upon treatment. Hou et al. [18] prepared 0.1 mm thick PLLA films via solvent casting. They treated the PLLA films with 1 mol/L NaOH for one hour, then rinsed with 0.1 mol/L HCl and water, and coupled gelatin to the surface via carbodiimide coupling. Gelatin modification was only successful after NaOH treatment and longer immersion time led to more gelatin on the surface.

Jaidev and Chatterjee [19] printed PLA scaffolds and immersed them in 5.0 mol/L NaOH for one hour. After washing with water and drying, poly(ethylene imine) (PEI) was conjugated to the surface using carbodiimide coupling. The CAs decreased from 124° to 74° after NaOH treatment, the surface roughness increased from 0.092 µm to 0.251 µm and microscopy showed a smooth surface before and a rough surface with a lot of grooves and protrusions after the NaOH treatment. Compression tests showed no change between treated and untreated PLA scaffolds. Tham et al. [20,21] created PLA films via hotpress molding. The films were washed with ethanol and immersed in 0.01 and 1 mol/L aqueous NaOH solution between 3 and 96 h. Again, a decrease of the CA from 65° to 50° (0.01 mol/L NaOH) and to 15° (1 mol/L NaOH) was observed. Measurements of the intrinsic viscosity indicated some reduction of the PLA molecular weight and optical microscopy showed an increase of the surface roughness vs. time.

We have previously shown that calcium phosphate (CP), an important biomaterial [22,23,24], does not adhere to freshly printed PLA scaffolds. Consequently, the development of stable, CP-coated 3D printed PLA biomaterials is rather challenging [25,26,27]. This is unfortunate because PLA/CP composites would be prime candidates for implant materials with favorable properties.

As a result, we have developed an automated process for the co-deposition of a stable hydrogel/calcium phosphate hybrid layer that strongly attaches to a 3D printed PLA scaffold [25]. The initial step towards the fabrication of these hybrid surface coatings is sodium hydroxide etching. This step is crucial for successful attachment, but is so far poorly understood and there is not much quantitative information available (see above) [11,14,15,16,17,18,19,20,21].

The current article therefore provides in-depth insights into the effects of NaOH etching of freshly prepared 3D printed PLA scaffolds. In contrast, our previous article focused on the fabrication and properties of a stable biocompatible hybrid coating on the PLA scaffold once the etching step was complete. As a result, the two studies taken together provide a detailed insight of how a 3D printed PLA scaffold can be activated using a controlled process (this study) and then, how this PLA can further be modified with a biocompatible polymer/inorganic hybrid surface coating (our previous work) [25]. The current article therefore adds a wealth of new information on the first, crucial step of surface activation based on data from atomic force microscopy (AFM), scanning electron microscopy (SEM), mechanical analysis, as well as infrared (IR), nuclear magnetic resonance (NMR), and X-ray photoelectron spectroscopy (XPS) that have not previously been available. Overall, the current article therefore enhances the understanding of the first step of surface activation and coating and thus adds valuable new insights to the previous study and the PLA biomaterials field in general.

## 2. Materials and Methods

*Materials*. Natural PLA filament (transparent, without any additives) was purchased from Ultimaker (PLA natural, diameter 2.85 mm, Ultimaker, Utrecht, The Netherlands). Sodium hydroxide was purchased as 5 mol/L volumetric standard solution from Carl Roth (Carl Roth, Karlsruhe, Germany). Ultrapure water was obtained from a PureLab Flex 4 (Elga LabWater, High Wycombe, UK) or a Simplicity UV (Merck, Darmstadt, Germany) setup. All ultrapure water had a resistivity of 18.2 MΩ∙cm at 25 °C.

*3D printing*. Standardized PLA scaffolds (square of 15.0 mm × 15.0 mm with a holding element 5.0 mm × 15.0 mm and a thickness of 1.0 mm) were printed on an Ultimaker 3 (Ultimaker, Utrecht, The Netherlands) 3D printer via fused deposition modelling with Cura Software 3.4.1. Nozzle diameter was 0.4 mm, layer height was 0.10 mm (first layer height was 0.27 mm), printing temperature was 205 °C, build plate temperature was 80 °C, and print speed was 70 mm/s. After printing, samples were dried overnight at 40 °C and weighed.

*Alkaline Treatment*. PLA scaffolds (each 0.341 ± 0.009 g) were treated with 10 mL of a 0.1, 0.2, 0.5, 1.0, 2.0, 3.0, or 5.0 mol/L NaOH solution for 1, 2, 5, 10, 30, 60, 120, 240, 480, 960, 1440, or 2880 min. After base treatment, the samples were washed with ultrapure water and dried overnight at 40 °C in air. Each experiment was performed three times. The 84 concentration-time-pairs resulted in a total of 252 alkaline treated samples (see Table 1 below).

*Calculation of erosion rate*. The erosion rate was calculated using a linear regression analysis of the relation of remaining mass after treatment vs. treatment time.

*Contact angle (CA) measurements*. The static CA was measured with a KSV Cam 100 (KSV Instruments, Helsinki, Finland) at room temperature with a Hamilton 1001 LT syringe (Hamilton, Reno, NV, USA). Drop size was 2 µL. On both sides of the scaffolds (printing bed side and printing nozzle side), three separate drops of ultrapure water were placed and the CA was measured parallel and perpendicular to the printing direction after rotating the scaffold by 90 °. Evaluation of the CAs was done with the Cam 100 software version 2.1.1.

*Attenuated Total Reflection Fourier Transform Infrared Spectroscopy (ATR-FTIR)*. ATR-FTIR was done on a Nicolet iS5 with iD7 ATR unit and XR diamond crystal purged with nitrogen or on a Nicolet iS10 FT-IR (Thermo Scientific, Waltham, MA, USA). Measurements were performed between 400 and 4000 cm^−1^ with 64 Scans and a resolution of 1 cm^−1^. Spectra were analyzed with the Omnic 9 Software.

*X-ray photoelectron spectroscopy (XPS)*. XPS measurements were done on a Prevac X-ray photoelectron spectrometer equipped with a monochromatized aluminium source Al-Kα (E = 1486.6 eV) and a hemispherical VG Scienta R3000 analyzer (Prevac, Rogów, Poland). Samples were introduced through a load lock into an ultrahigh vacuum analytical chamber with a base pressure of 5∙10^−9^ mbar. To compensate the charge on the surface of nonconductive samples, a low energy electron flood gun (FS40A-PS, Henniker Scientific, Runcorn UK) was used. Data analysis was performed via Origin 2018.

*Scanning Electron Microscopy (SEM)*. SEM images were taken with a Phenom Pro operated at 5 kV equipped with a backscatter electron detector and a sample holder for charge reduction (Phenom World, Thermo Fisher Scientific, Waltham, MA, USA). Samples were sputtered with a 10 nm gold layer using a 208 HR sputter coater (Cressington, Watford, UK). SEM images of the sample cross sections were taken with a JSM-6510 operated at 5 kV (JEOL, Akishima, Japan). Samples were sputtered with 10 nm gold/palladium using a SC7620 Mini Sputter Coater (Quorum Technologies, Lewes, UK).

*Force spectroscopy and Atomic Force Microscopy (AFM)*. Characterization of mechanical and structural properties by force spectroscopy was done on a Bruker Dimension Icon working in PeakForce Tapping mode using triangular silicon nitride cantilevers (ScanAssist-Air; nominal spring constant equal to 0.4 N/m) (all Bruker, Billerica, MA, USA). AFM images were collected on a FlexAFM (Nanosurf AG, Liestal, Switzerland) in tapping mode using beam shaped soft-tapping cantilevers (HQ:NSC14/Al BS, nominal spring constant 5 N/m) with Nanosurf C 3000 software. Data analysis was done with the Gwyddion 2.55 software. Roughness was calculated as root means square (RMS) roughness from 5 × 5 µm areas.

*Gel permeation chromatography (GPC)*. GPC with simultaneous UV and refractive index detection was conducted with tetrahydrofuran as eluent (flow rate: 0.5 mL/min) at room temperature. The stationary phase was a 300 × 8 mm^2^ PSS SDV linear M column (particle size: 3 µm, molar mass range: 10^2^ to 10^6^ Da). Solutions contained between 0.1 and 0.2 % polymer (1.5 mg to 2.0 mg) and were filtered through 0.45 µm filters prior to measurements. The injected volume was 100 µL. Polystyrene standards were used for calibration. Column and polystyrene standards were both from PSS Polymer Standards Service (Mainz, Germany).

*Tensile Testing*. Measurements were done on a Universal Test System using an MTS Exceed Model E42.503 (MTS Systems, Eden Prairie, MN, USA). A test program based on a standard template from the MTS Software was used and test velocity was set to 1 mm/s. Specimen width and thickness were measured for each sample separately before each measurement. Data analysis was done with the TW Essentials 4.3.0.352 software and the E-modulus was calculated from strength and travel path.

*Differential scanning calorimetry (DSC)*. DSC measurements were done on a DSC 214 Polyma (Netzsch, Selb, Germany) from 40 to 180 °C at a heating rate of 1 K/min. Data analysis was done with Proteus Analysis 7.0.1.

*Nuclear magnetic resonance (NMR) spectroscopy*. ^1^H and ^13^C NMR spectroscopy was done on a Bruker Avance 500 MHz spectrometer (Bruker, Billerica, MA, USA). CDCl_3_ was used as solvent and 16 scans were recorded for ^1^H and 256 scans for ^13^C spectra. The CHCl_3_ peak at 7.26 ppm was used for calibration of the ^1^H-NMR spectra and CHCl_3_ peak at 77 ppm was used for calibration for the ^13^C-NMR spectra.

## 3. Results

**Figure 1:**
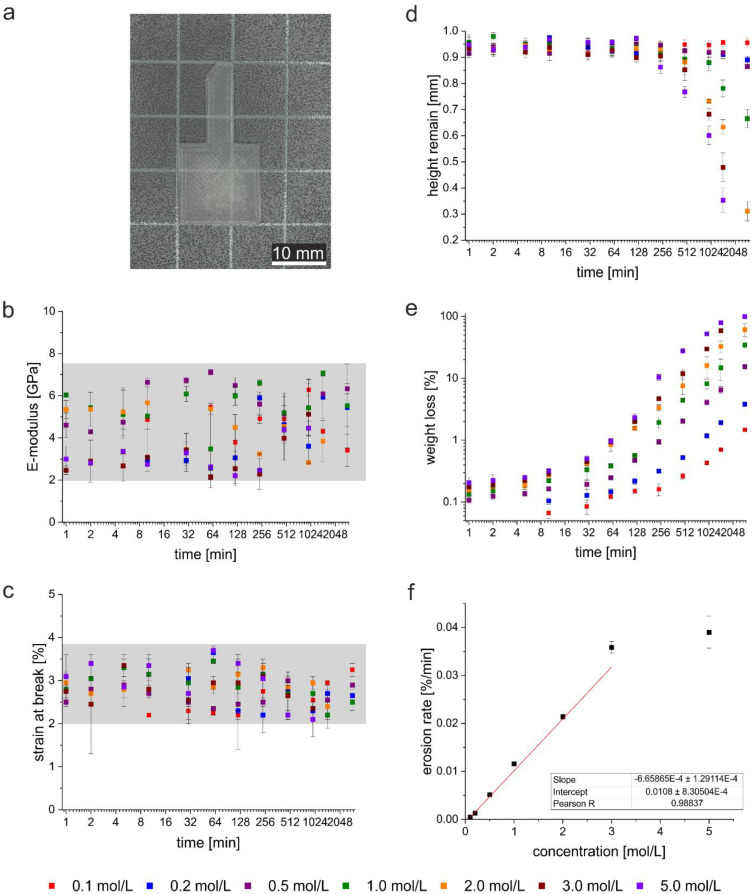
(**a**) Photograph of a typical sample used, (**b**) elastic modulus, (**c**) strain at break vs. exposure time and NaOH concentration, (**d**) remain height, (**e**) weight loss, (**f**) rate of erosion with linear regression. Grey areas indicate region where experimental E-moduli and strain at break have been measured. Note that for NaOH concentrations of 3.0 and 5.0 mol/L and exposure times of 1440 min or longer no data are available due to complete sample dissolution. As a result, for these materials and treatments, no data can be plotted.

As detailed above, the 3D printed PLA scaffolds were treated with different NaOH solutions for different times (materials and methods section) and the properties of the treated samples are compared to the untreated starting materials. Figure 1 summarizes the results vs. exposure times and NaOH concentration.

Figure 1a shows a typical PLA print used for the experiments. The printed objects are white, semitransparent solids with a handle to hold (some photos of the finished surface are shown in Figure A5). Figure 1b shows the E-modulus vs. sample treatment and Figure 1c shows the strain at break of the materials. Overall the data are quite scattered but remain within the same, rather broad, range of properties throughout the entire treatment and measurement range. Therefore, Figure 1b and c show no significant change in the mechanical properties vs. sample treatment.

Figure 1d shows the reduction of the sample thickness upon treatment. Within the first hour, the thickness changes are below 1%. For NaOH concentrations up to 0.2 mol/L, no height changes are observed even at extended periods of time. With higher NaOH concentrations, the rate of degradation increases and there is a clear correlation with the NaOH concentration. After 48 h and NaOH concentrations of 3.0 mol/L and higher, the PLA scaffolds dissolve completely while at lower NaOH concentrations and shorter immersion time, the scaffold remains intact but is affected by the hydrolytic process.

Figure 1e shows the mass loss vs. exposure time and NaOH concentration. Comparison with the data shown in Figure 1d reveal correlations between treatment times and NaOH concentrations but also two interesting differences between dimensional changes (Figure 1d) and weight loss (Figure 1e): while low NaOH concentrations do not lead to a change in the dimensions, Figure 1e reveals that the same NaOH concentrations lead to a weight loss at extended times. Similarly, the weight losses observed at higher NaOH concentrations are apparent at much earlier times than the dimensional changes in Figure 1d. Already after 10 min, the first weight loss is observed when treated with high NaOH concentrations while at the same time, no change in the dimensions are visible yet.

From the data shown in Figure 1d and e, the erosion rate was determined (see experimental section). Figure 1f shows that the erosion rate increases linearly up to a NaOH concentration of 3.0 mol/L. Samples treated with 5.0 mol/L NaOH show a rather significant deviation from linearity.

FDM 3D printing leads to scaffolds with different surfaces. Depending on whether the side is in contact with the glass build plate or is oriented towards the printing nozzle, the surface is either smooth (glass support side) or rough (printing side). To enable a good comparison of the data, only the smooth side of the printed scaffolds was used for further analysis.

**Figure 2:**
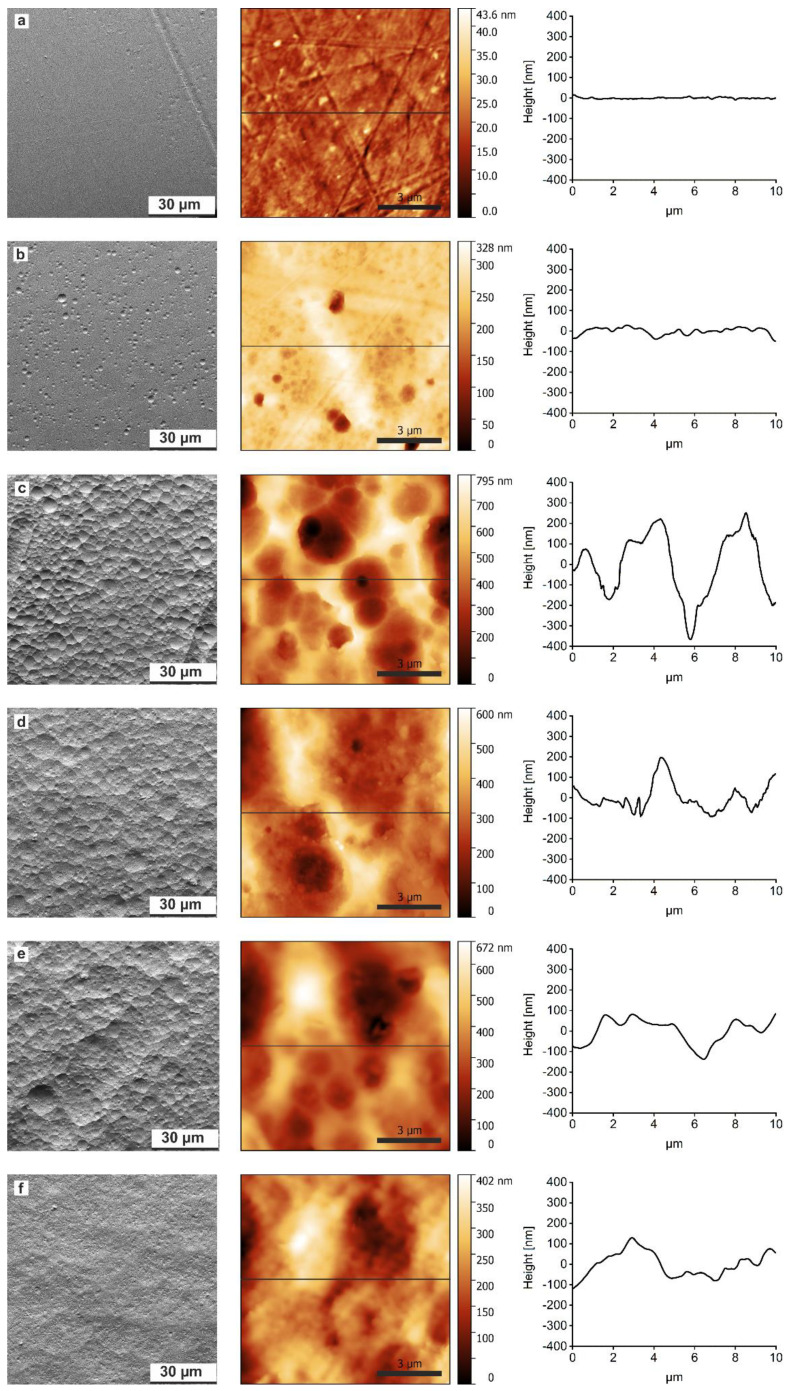
SEM (left column) and AFM (middle column) images with representative cross-sections (right column) of PLA: (**a**) untreated sample, (**b**) treatment with 1.0 mol/L NaOH for 120 min (2 h), (**c**) 240 min (4 h), (**d**) 480 min (8 h), (**e**) 960 min (16 h), and (**f**) 2880 min (48 h).

Figure 2 shows representative scanning electron microscopy (SEM) images of a sample treated with 1.0 mol/L NaOH vs. time. The freshly printed untreated sample exhibits an essentially flat surface with very few and shallow surface features (grooves, wells, and indentations). After two hours, the number of indentations and wells increases and the SEM images suggest that they are deeper than in the initial materials but their diameter is still rather small on the order of 0.5–4 µm. Longer treatment (4 h) leads to interconnection of the wells, also their number and diameter increases. After 8 h the surface is characterized by a smoothing of pointed edges along the indentations. At even longer times, the surface appears more rounded with less pronounced edge features. The diameters of the features increase to ca. 5 to 15 µm. Moreover, at this stage, another generation of smaller pits or hole-like features appears.

**Figure 3:**
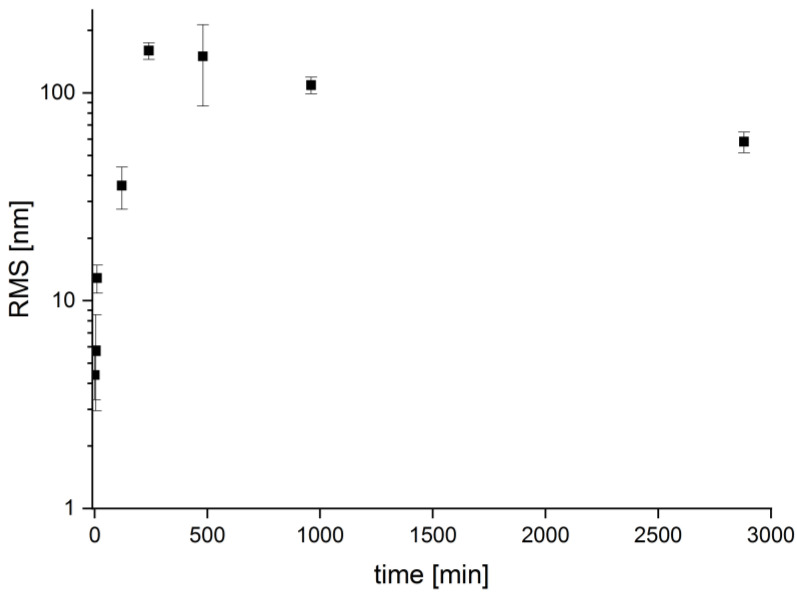
Surface roughness vs. treatment time for a 1.0 mol/L NaOH treatment. Note that the ordinate is on a log10 scale. RMS is root mean square roughness.

These qualitative investigations are supported by atomic force microscopy (AFM). AFM images confirm the SEM images, Figure 2, and also show the formation of larger and rougher features with longer reaction times. Moreover, AFM reveals an interesting observation: initially the surface roughness (root mean square roughness, RMS) of the printed material is quite low at 4.4 ± 1.1 nm but significantly increases to 160 ± 15 nm until 4 h of treatment, due to surface erosion. [28] Thereafter, the surface roughness decreases again, albeit not to its original, very low surface roughness, Figure 3. Table 1 summarizes the results from scanning electron microscopy on a qualitative level. Three different terms are used to describe the surface: smooth, rough, and rounded. The smooth surface is flat overall without significant indentations, Figure 2a. Rough surfaces show a lot of features (grooves, wells, and indentations) with varying size and depth, Figure 2b–e. A rounded surface appears less edgy with rounder and less pronounced features, Figure 2f.

**Figure 4:**
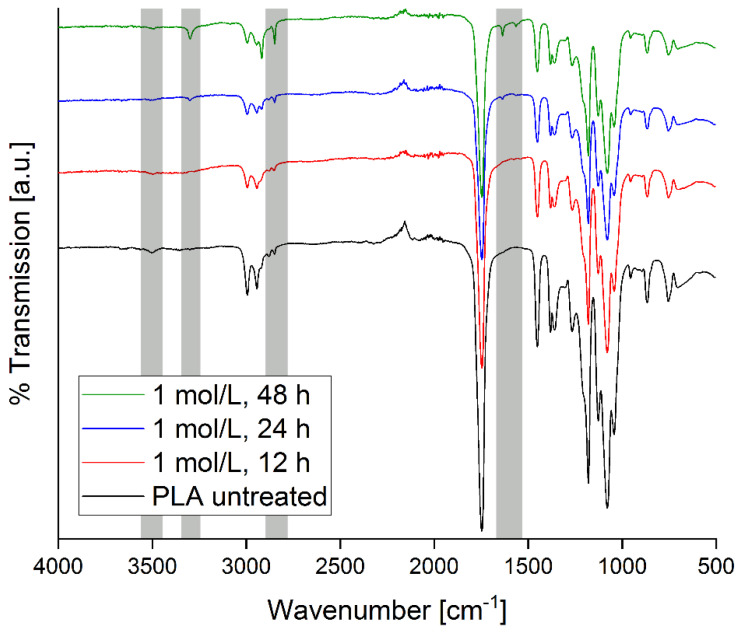
IR-Spectra obtained from untreated PLA and from PLA treated with 1.0 mol/L NaOH for 12, 24, and 48 h. Grey areas indicate regions of most important spectral changes.

Further analysis was done with nuclear magnetic resonance (^1^H-NMR, ^13^C-NMR) and attenuated total reflection Fourier transform infrared (ATR-FTIR) spectroscopy. Regular IR spectroscopy is not very sensitive to surface changes, so chemical changes that only affect the surface are hard to distinguish from the background. However, the IR spectra of the PLA treated with higher concentrations and at longer reaction times exhibit clear changes, Figure 4. For example, spectra of samples treated with 1.0 mol/L NaOH show new bands after 24 h that become more intense after 48 h while other bands become weaker or disappear. Generally all spectra show identical features upon base treatment at longer times.

A band at 3500 cm^−1^ can be assigned to O-H stretch vibrations. It becomes weaker upon treatment. A new band at 3300 cm^−1^ assigned to O-H valence vibration appears and becomes gradually more intense with longer treatment time. Bands at 2995 and 2945 cm^−1^ decrease in intensity while bands at 2918 and 2850 cm^−1^ increase in intensity. They all are assigned to symmetric and asymmetric C-H stretch vibrations. There is a slight decrease in intensity of the carbonyl bands at 1748 and 1452 cm^−1^. Additional bands at 1636 and 1566 cm^−1^ appear upon treatment; they can be assigned to newly formed free carbonyl and hydroxyl groups.

Several bands show only a very slight loss of intensity without any significant change in shape or a wavenumber shift: they are at 1453 cm^−1^ (CH_3_ deformation vibration), at 1381, 1360, and 1267 cm^−1^ (CH deformation vibration), at 1180, 1130, and 1080 cm^−1^ (C-O stretch vibration), at 1041 cm^−1^ (O-H deformation vibration), and finally at 955, 869, and 755 cm^−1^ (C-C stretch vibration). The vibration at 869 cm^−1^ is from amorphous PLA and the vibration at 755 cm^−1^ is from crystalline PLA [1]. Both bands are clearly visible, consistent with the semicrystalline nature of the PLA used in this study.

**Figure A1:**
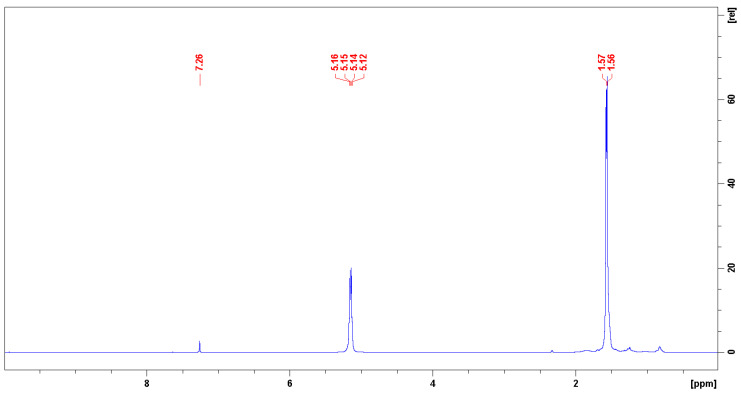
1H NMR spectra of untreated PLA.

**Figure A2:**
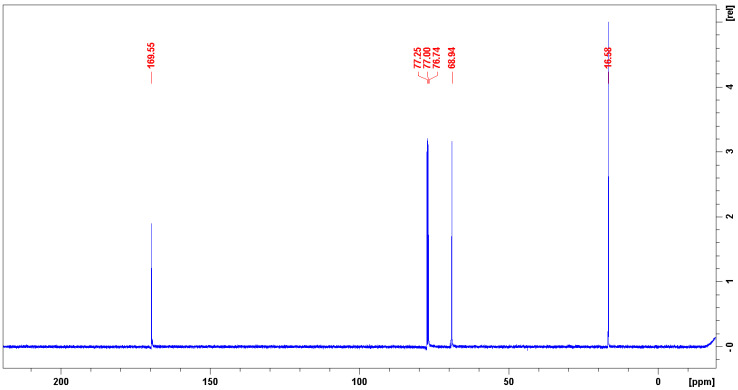
13C NMR spectra of untreated PLA.

**Figure A3:**
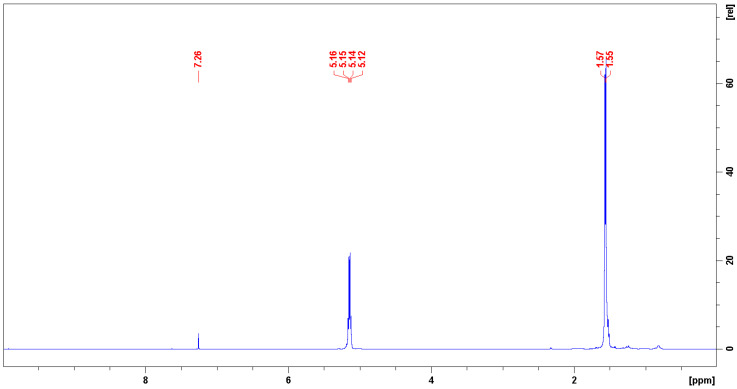
1H NMR spectra of PLA with 5 mol/L 8 h.

**Figure A4:**
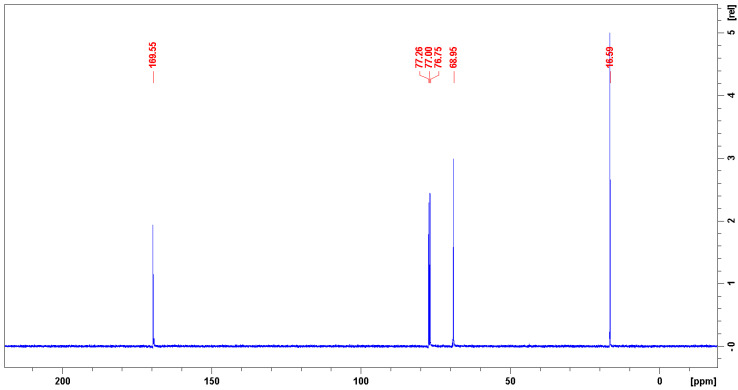
13C NMR spectra of PLA with 5 mol/L 8 h.

**Figure A5:**
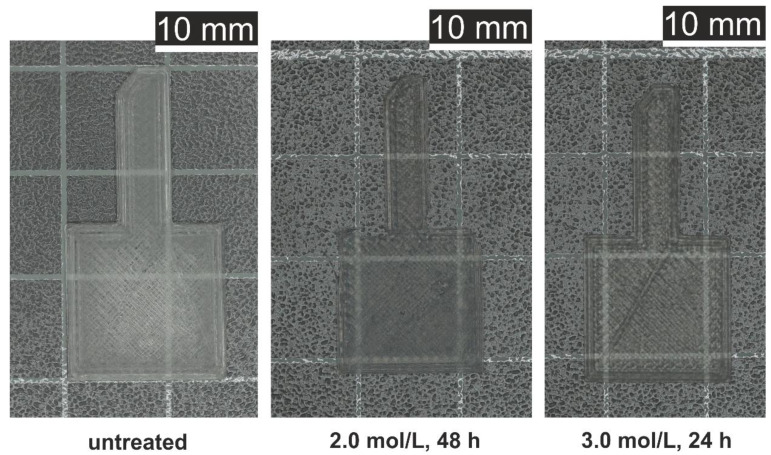
PLA surface of different samples.

Similarly, ^1^H-NMR and ^13^C-NMR spectra (Figure A1, Figure A2, Figure A3 and Figure A4) obtained from PLA dissolved in CDCl_3_ after NaOH treatment do not show changes in the chemical structure of the PLA compared to the starting materials. Peaks from the free carbonyl and carboxyl groups which could be expected from ester cleavage upon NaOH treatment cannot be observed.

**Figure 5:**
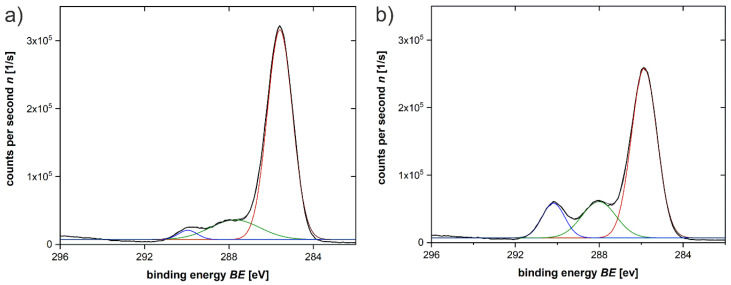
XPS spectra of the PLA: (**a**) untreated, (**b**) treated with 1.0 mol/L NaOH for 8 h.

In contrast, X-ray photoelectron spectroscopy (XPS) data show an increase in the carbonyl signal after treatment of the PLA scaffold with 1.0 mol/L NaOH for 8 h. There is a clear increase of the contribution of the band characteristic of C=O (290 eV) and of the C–OH (287 eV) band in the whole XPS spectrum after the treatment, Figure 5.

Additional information about surface properties can be obtained from contact angle (CA) measurements. The CA depends on the chemical and geometrical properties of a surface and the printing process therefore has a tremendous influence on the CA.

**Figure 6:**
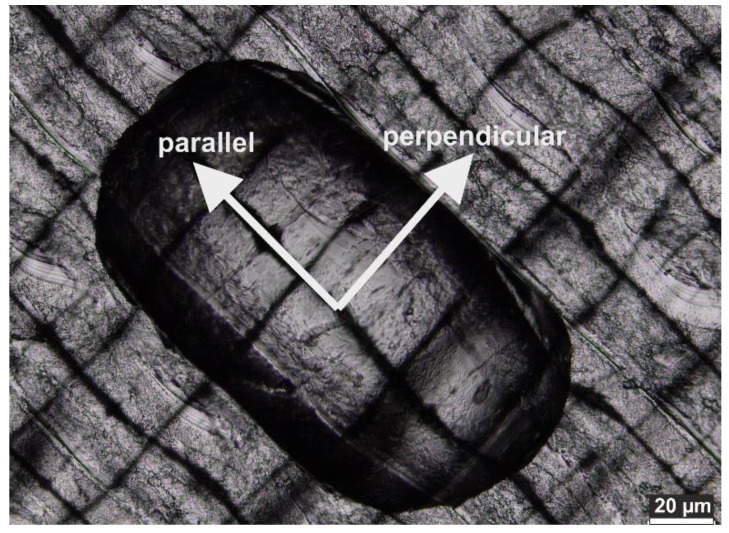
Anisotropic shape of water drop on a 3D printed surface. The terms “parallel” and “perpendicular” indicate drop orientation with respect to printing direction.

It is important to state here that there is also an effect of the PLA alignment during printing: the scaffolds are printed layer-by-layer and the water drop orients parallel to the printing layers. This results in an anisotropic drop shape, Figure 6, and two different contact angles parallel and perpendicular to the printing direction. There is a fairly large difference between these two CAs and the CAs measured parallel to the printing direction are always higher. As a result, the CA needs to be measured before and after NaOH treatment for both the parallel and perpendicular orientation.

**Figure 7:**
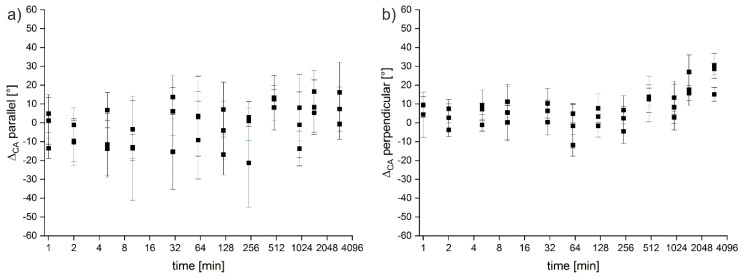
Changes in the CA (Δ_CA_) on PLA after treatment with 1.0 mol/L NaOH for different times. (**a**) Δ_CA_ parallel to the printing direction, (**b**) Δ_CA_ perpendicular to the printing direction. Data (not shown) for the other materials and treatments are very similar. Note the Log2 scale of the x-axis chosen for better clarity of representation.

Table 2 summarizes the CAs obtained from the untreated samples and Figure 7 shows the change in CA of all samples treated with 1.0 mol/L of NaOH vs. the untreated starting material (that is Δ_CA_ = CA_untreated_ − CA_treated_). Similar to the E-modulus and the strain at break, Figure 1, the CA data are scattered over a certain range and while there appears a moderate change of the CA between untreated and treated materials, the differences are rather low and only reach a ca. 30° difference. The CAs obtained from the parallel orientation do not show a trend at all. In contrast, the CA obtained from the perpendicular direction indicates a slight CA increase but again, the CAs are distributed over a rather large range.

Finally, differential scanning calorimetry (DSC) data (not shown) show no change in the materials upon treatment. All materials, freshly printed and treated with NaOH, show the exact same DSC traces identical to data shown previously [25]. In the heating curves, a glass transition at 58 °C, a cold crystallization at 102 °C, and a double melting transition at 146 and 154 °C without completely separated melting peaks are observed. Upon cooling, only a glass transition around 55 °C is observed but no crystallization.

## 4. Discussion

As stated in the introduction, the tuning of PLA surfaces is an important point in biomaterials application. The focus of the current study is on the detailed evaluation of the effects of a very simple NaOH treatment. The data can clearly be divided in two groups: some data indicate no or only small changes of the material upon treatment (DSC, E-modulus, strain at break) while other data clearly show that there is a change in the materials (weight loss, dimensional changes, erosion rate, IR, SEM, AFM, XPS). CA measurements are somewhat in between the two groups.

While initially these two groups of data seem different, it is in fact quite straightforward to reconcile the results. Data of the first group are from methods that probe the bulk PLA. Clearly these data show that the bulk structure is not directly affected by the treatment. This is consistent with literature [10,16,21]. The E-modulus is around 4.5 ± 1.6 GPa, which is higher than reported in the literature [1,29,30,31] but the same publications also state that here is a certain variation to these values. Because of our unique sample design, tensile testing cannot follow ISO or ASTM standard procedures. As a result, the absolute values must not be generalized for any PLA, but nevertheless, the experimental values clearly demonstrate that the treatment has no effect on the E-modulus of the scaffolds.

In contrast the data from the other group of experiments are from methods that essentially probe the surface. Especially ATR-IR, SEM, AFM, and XPS are surface analysis tools and the respective data clearly prove that the treatment of PLA scaffolds with NaOH leads to a surface modification that can be controlled by the exposure time and the NaOH concentration.

Indeed, Mohd Sabee et al. [11] have already shown that NaOH treatment leads to a change in the surface characteristics as shown by CA measurements. However, neither these authors nor Argentati et al. [14] observed a chemical change in their materials. This is qualitatively consistent with the current materials treated at shorter treatment times. At short exposure times, also here, neither IR nor NMR spectroscopy show any changes. However, our IR and XPS data clearly show that longer treatments lead to chemical modification, likely by ester cleavage and the formation of –OH and –COOH groups on the PLA surface. We currently speculate that although there is no direct spectroscopic evidence the ester cleavage starts rather early on but that there need to be a certain number of pendant –OH and –COOH groups on the PLA surface to become visible in the IR or XPS. Indeed the IR spectra show an increasing intensity of the respective bands vs. treatment and XPS provides evidence of hydrolysis vs. reaction time [21].

Because of their rather broad variation, the CAs data are more difficult to evaluate. Consistent with literature, the CA depends on both the chemical properties and the surface roughness. The two common models for describing the wettability of surfaces are by Wenzel and Cassie and Baxter [32,33,34,35]. However, these models are for idealized surfaces and have their limitations in the description of real surfaces [35]. Mukherjee et al. [36] described a model of the contact angle of different surfaces and the influence of roughness and chemical properties. Their data are consistent to our observations that roughness increases the CA and a more surface with stronger chemical interactions between surface and water leads to lower CA. Clearly, the current surfaces exhibit a combination of effects and it is therefore hardly possible to assign either of these models to a description of the behavior.

In spite of this, there are a few precedents that are useful for assessing the effects observed here. Nakae et al. [34] described the effects of surface roughness on the wettability and first found an increase and later a decrease of the CA, consistent with our data, Figure 2. Ma et al. [33] explained the wettability after changing the surface structure without chemical altering with either the Wenzel or the Cassie-Baxter theory. In contrast, Kubiak at al. [32] suggested that the Wenzel model is suited for use on hydrophilic surfaces and the Cassie-Baxter model for hydrophobic surfaces. Again, as the current materials are between both models, it is rather difficult to properly assign a specific model for a description of the CA.

As the surfaces studied here (like most surfaces) simultaneously change in their roughness and their chemical composition, the CA changes cannot be explained only by roughness changes. Rather they likely stem from a combination of increased CA by roughness and decreased CA by chemical changes. Presumably, both of these changes start early on in the treatment process. The cleavage of the ester bonds (which is the factor contributing to a higher hydrophilicity as the reaction proceeds) always goes with material erosion (which leads to higher roughness and presumably to an increasing hydrophobicity).

Overall, the data clearly show that a systematic treatment of PLA with NaOH provides access to a variety of surfaces that may be interesting for further functionalization and use. Clearly there are several parameters that play a role in the definition of the surface properties: a change in the surface morphology, surface roughness, and surface chemistry will all affect, for example, the CA or the possibility to further chemically functionalize the surface. Although the surface as a whole is not more hydrophilic after the treatment, the activation of the surface provides functional groups for further reactions and it also provides an interesting tuneability of the surface roughness. In contrast to the quite drastic chemical and morphological changes of the surface, the bulk PLA appears largely unaffected by the treatment. This is consistent with previous studies [10,11,14,15,16,19,21] and is clearly reflected by the invariance of, e.g., the mechanical or thermal properties vs. sample treatment.

## 5. Conclusions

NaOH treatment is a simple yet powerful method for activating 3D printed PLA surfaces. The current study provides the most complete dataset on NaOH treatment of 3D printed PLA scaffolds so far. The data clearly show that suitable treatments will leave the bulk PLA essentially unaltered while providing controllable changes or adaptations to the PLA surface. The NaOH cleaves the ester bonds to provide numerous hydrophilic groups on the surface, while erosion at the same time leads to significant changes of the surface roughness. The combination of both leads to the final surface properties, which depend on NaOH concentration and treatment time and is therefore easy adjustable. A seemingly simple process like NaOH etching is therefore powerful and can precisely be adjusted to design a specific surface morphology or surface chemistry while maintaining the overall structural integrity of the 3D printed PLA scaffold.

## Figures and Tables

**Table 1:** Surface morphology of PLA scaffolds treated with NaOH. The surface is qualitatively categorized as described above. “Degraded” refers to samples that are mostly dissolved and cannot be analyzed.

	Concentration [mol/L]	0.1	0.2	0.5	1.0	2.0	3.0	5.0
Time [min]								
1		smooth	smooth	smooth	smooth	smooth	smooth	smooth
2		smooth	smooth	smooth	smooth	smooth	smooth	smooth
5		smooth	smooth	smooth	smooth	smooth	smooth	smooth
10		smooth	smooth	smooth	smooth	smooth	smooth	smooth
30		smooth	smooth	smooth	smooth	smooth	smooth	rough
60 (1 h)		smooth	smooth	smooth	smooth	rough	rough	rough
120 (2 h)		smooth	smooth	rough	rough	rough	rough	rough
240 (4 h)		smooth	smooth	rough	rough	rough	rough	rough
480 (8 h)		rough	rough	rough	rough	rough	rough	rounded
960 (16 h)		rough	rough	rough	rough	rough	rounded	rounded
1440 (24 h)		rough	rough	rounded	rounded	rounded	rounded	rounded
2880 (48 h)		rough	rounded	rounded	rounded	rounded	degraded	degraded

**Table 2:** Contact Angle (CA) of 3D printed PLA without treatment.

	**Parallel Direction**	**Perpendicular Direction**
Mean CA	98.0° ± 15.0°	69.0° ± 5.7°
Lowest CA (minimum)	60.4°	46.6°
Highest CA (maximum)	119.4°	84.9°

## Appendix A

NMR spectra of untreated PLA and a PLA treated with 5 mol/L NaOH for 8 h.

## References

[1] Garlotta D. (2001). A Literature Review of Poly(Lactic Acid). J. Polym. Environ..

[2] Xiao L., Wang B., Yang G., Gauthier M. (2006). Poly(Lactic Acid)-Based Biomaterials: Synthesis, Modification and Applications. Biomed. Sci. Eng. Technol..

[3] Spinu M., Jackson C., Keating M.Y., Gardner K.H. (1996). Material Design in Poly(Lactic Acid) Systems: Block Copolymers, Star Homo- and Copolymers, and Stereocomplexes. J. Macromol. Sci. Part A.

[4] Masutani K., Kimura Y. (2014). Chapter 1. PLA Synthesis. From the Monomer to the Polymer. Poly(lactic acid) Science and Technology: Processing, Properties, Additives and Applications.

[5] Farah S., Anderson D.G., Langer R. (2016). Physical and mechanical properties of PLA, and their functions in widespread applications—A comprehensive review. Adv. Drug Deliv. Rev..

[6] Gordeev E.G., Degtyareva E.S., Ananikov V.P. (2016). Analysis of 3D printing possibilities for the development of practical applications in synthetic organic chemistry. Russ. Chem. Bull..

[7] Ambrosi A., Pumera M. (2016). 3D-printing technologies for electrochemical applications. Chem. Soc. Rev..

[8] Rebelo R., Fernandes M., Fangueiro R. (2017). Biopolymers in Medical Implants: A Brief Review. Procedia Eng..

[9] Przekora A. (2019). Current trends in fabrication of biomaterials for bone and cartilage regeneration: Materials modifications and biophysical stimulations. Int. J. Mol. Sci..

[10] Cao Y., Croll T.I., Cooper-White J.J., O’Connor A.J., Stevens G.W. (2004). Production and Surface Modification of Polylactide-Based Polymeric Scaffolds for Soft-Tissue Engineering. Biopolymer Methods in Tissue Engineering.

[11] Mohd Sabee M.M.S., Kamalaldin N.A., Yahaya B.H., Abdul Hamid Z.A. (2016). Characterization and in vitro study of surface modified PLA microspheres treated with NaOH. J. Polym. Mater..

[12] Ajiko M., Enomoto K., Suzuki K., Yamaguchi A. (1995). Basic Properties of Polylactic Acid Produced by the Direct Condensation Polymerization of Lactic Acid. Bull. Chem. Soc. Jpn..

[13] Nofar M., Park C.B. (2014). Poly (lactic acid) foaming. Prog. Polym. Sci..

[14] Argentati C., Morena F., Montanucci P., Rallini M., Basta G., Calabrese N., Calafiore R., Cordellini M., Emiliani C., Armentano I. (2018). Surface hydrophilicity of poly(L-lactide) acid polymer film changes the human adult adipose stem cell architecture. Polymers.

[15] Inagaki N., Narushima K., Tsutsui Y., Ohyama Y. (2002). Surface modification and degradation of poly(lactic acid) films by Ar-plasma. J. Adhes. Sci. Technol..

[16] Croll T.I., O’Connor A.J., Stevens G.W., Cooper-White J.J. (2004). Controllable Surface Modification of Poly(lactic- c o -glycolic acid) (PLGA) by Hydrolysis or Aminolysis I: Physical, Chemical, and Theoretical Aspects. Biomacromolecules.

[17] Zhao K., Yang X., Chen G.Q., Chen J.C. (2002). Effect of lipase treatment on the biocompatibility of microbial polyhydroxyalkanoates. J. Mater. Sci. Mater. Med..

[18] Hou X., Zhang B.-I., She F., Cui Y.-I., Shi K., Yao K. (2003). Surface of Gelatine Modified Poly(L-Lactic Acid) Film. Chinese J. Polym. Sci..

[19] Jaidev L.R., Chatterjee K. (2019). Surface functionalization of 3D printed polymer scaffolds to augment stem cell response. Mater. Des..

[20] Tham C.Y., Abdul Hamid Z.A., Ahmad Z., Ismail H. (2014). Surface Modification of Poly(lactic acid) (PLA) via Alkaline Hydrolysis Degradation. Adv. Mater. Res..

[21] Abdul Hamid Z.A., Tham C.Y., Ahmad Z. (2018). Preparation and optimization of surface-engineered poly(lactic acid) microspheres as a drug delivery device. J. Mater. Sci..

[22] Dorozhkin S.V. (2009). Calcium orthophosphates in nature, biology and medicine. Materials.

[23] Weiner S. (2003). An Overview of Biomineralization Processes and the Problem of the Vital Effect. Rev. Mineral. Geochem..

[24] Habraken W., Habibovic P., Epple M., Bohner M. (2016). Calcium phosphates in biomedical applications: Materials for the future?. Mater. Today.

[25] Schneider M., Günter C., Taubert A. (2018). Co-deposition of a hydrogel/calcium phosphate hybrid layer on 3D printed poly(lactic acid) scaffolds via dip coating: Towards automated biomaterials fabrication. Polymers.

[26] Bleek K., Taubert A. (2013). New developments in polymer-controlled, bioinspired calcium phosphate mineralization from aqueous solution. Acta Biomater..

[27] Schweizer S., Taubert A. (2007). Polymer-Controlled, Bio-Inspired Calcium Phosphate Mineralization from Aqueous Solution. Macromol. Biosci..

[28] Göpferich A. (1996). Mechanisms of polymer degradation and erosion1. Biomater. Silver Jubil. Compend..

[29] Pastor-Artigues M.M., Roure-Fernández F., Ayneto-Gubert X., Bonada-Bo J., Pérez-Guindal E., Buj-Corral I. (2020). Elastic asymmetry of PLA material in FDM-printed parts: Considerations concerning experimental characterisation for use in numerical simulations. Materials.

[30] Zhao Y., Chen Y., Zhou Y. (2019). Novel mechanical models of tensile strength and elastic property of FDM AM PLA materials: Experimental and theoretical analyses. Mater. Des..

[31] Lopez D.M.B., Ahmad R. (2020). Tensile mechanical behaviour of multi-polymer sandwich structures via fused deposition modelling. Polymers.

[32] Kubiak K.J., Mathia T.G. (2014). Anisotropic Wetting of Hydrophobic and Hydrophilic Surfaces–Modelling by Lattice Boltzmann Method. Procedia Eng..

[33] Ma C., Bai S., Peng X., Meng Y. (2013). Anisotropic wettability of laser micro-grooved SiC surfaces. Appl. Surf. Sci..

[34] (1998). Hideo Nakae; Ryuichi Inui; Yosuke Hirata; Hiroyuki Saito Effects of surface roughness on wettability. Acta Mater..

[35] Quéré D. (2002). Rough ideas on wetting. Phys. A Stat. Mech. Its Appl..

[36] Mukherjee S., Martínez-González J.A., Dowling D.P., Gowen A.A. (2018). Predictive modelling of the water contact angle of surfaces using attenuated total reflection-Fourier transform infrared (ATR-FTIR) chemical imaging and partial least squares regression (PLSR). Analyst.

